# Effects of Fear

**Published:** 1858-04

**Authors:** 


*Effects of Fear.*—A Parisian Physician, during his visits made in a hired fly, had received a bottle of real Jamaica rum as a sample, but found, after returning home, that he had left it in the carriage. He went to the office, and informed the manager that he had left a virulent poison in one of the carriages, and desired him to prevent any of the coachmen from drinking it. Hardly had he got back when he was summoned in gaeat haste to three of these worthies, who were suffering from the most horrible colic, and great was his difficulty in persuading them that they had only stolen some most excellent rum.—*Va. Med. Journal.*

